# Transcriptome analysis of *Bupleurum chinense *focusing on genes involved in the biosynthesis of saikosaponins

**DOI:** 10.1186/1471-2164-12-539

**Published:** 2011-11-02

**Authors:** Chun Sui, Jie Zhang, Jianhe Wei, Shilin Chen, Ying Li, Jiesen Xu, Yue Jin, Caixiang Xie, Zhihui Gao, Hongjiang Chen, Chengmin Yang, Zheng Zhang, Yanhong Xu

**Affiliations:** 1 Institute of Medicinal Plant Development (IMPLAD), Chinese Academy of Medical Sciences & Peking Union Medical College, No. 151, Malianwa North Road, Haidian District, Beijing 100193, China

## Abstract

**Abstract:**

**Background**

*Bupleurum chinense *DC. is a widely used traditional Chinese medicinal plant. Saikosaponins are the major bioactive constituents of *B. chinense*, but relatively little is known about saikosaponin biosynthesis. The 454 pyrosequencing technology provides a promising opportunity for finding novel genes that participate in plant metabolism. Consequently, this technology may help to identify the candidate genes involved in the saikosaponin biosynthetic pathway.

**Results**

One-quarter of the 454 pyrosequencing runs produced a total of 195, 088 high-quality reads, with an average read length of 356 bases (NCBI SRA accession SRA039388). A *de novo *assembly generated 24, 037 unique sequences (22, 748 contigs and 1, 289 singletons), 12, 649 (52.6%) of which were annotated against three public protein databases using a basic local alignment search tool (E-value ≤1e-10). All unique sequences were compared with NCBI expressed sequence tags (ESTs) (237) and encoding sequences (44) from the *Bupleurum *genus, and with a Sanger-sequenced EST dataset (3, 111). The 23, 173 (96.4%) unique sequences obtained in the present study represent novel *Bupleurum *genes. The ESTs of genes related to saikosaponin biosynthesis were found to encode known enzymes that catalyze the formation of the saikosaponin backbone; 246 cytochrome P450 (*P450*s) and 102 glycosyltransferases (*GT*s) unique sequences were also found in the 454 dataset. Full length cDNAs of 7 *P450*s and 7 uridine diphosphate *GT*s (*UGT*s) were verified by reverse transcriptase polymerase chain reaction or by cloning using 5' and/or 3' rapid amplification of cDNA ends. Two *P450*s and three *UGT*s were identified as the most likely candidates involved in saikosaponin biosynthesis. This finding was based on the coordinate up-regulation of their expression with *β-AS *in methyl jasmonate-treated adventitious roots and on their similar expression patterns with *β-AS *in various *B. chinense *tissues.

**Conclusions:**

A collection of high-quality ESTs for *B. chinense *obtained by 454 pyrosequencing is provided here for the first time. These data should aid further research on the functional genomics of *B. chinense *and other *Bupleurum *species. The candidate genes for enzymes involved in saikosaponin biosynthesis, especially the *P450*s and *UGT*s, that were revealed provide a substantial foundation for follow-up research on the metabolism and regulation of the saikosaponins.

## Background

*Bupleurum chinense *DC., a perennial herb native to China, belongs to the Umbelliferae family and the genus *Bupleurum *L. This herb is used worldwide for medicinal purposes, but is especially common in China, Japan, and South Korea [1]. In traditional Chinese medicine, the roots of *B. chinense *and other *Bupleurum *species are known as Chinese thorowax roots (*Radix bupleuri*), or "chaihu" in Chinese. For more than 2, 000 years these roots have been used for their anti-inflammatory, anti-pyretic, and anti-hepatotoxic effects in the treatment of common colds, fever, influenza, hepatitis, malaria, and menoxenia [2,3]. The major bioactive components of *Radix bupleuri *are the saikosaponins (SSs), which belong to the oleanane-type triterpene saponins. Although more than 75 monomer SSs have been isolated from Radix bupleuri [4,5], only SS-a, SS-b2, SS-c, and SS-d have been pharmacologically examined [6-10], because of the low SS content (usually ca. 1% *w*/*w *in dried roots) [11]. Different monomer SSs have been reported to exhibit different predominant pharmacological effects. For example, among the SSs isolated from *B. falcatum*, SS-a and SS-d, but not SS-c, have anti-inflammatory activities [12]. Whereas SS-c has no correlation with cell growth inhibition, other SSs can inhibit cell growth, as well as induce cancer cell differentiation and apoptosis. Hence, SS-c may have the potential for therapeutic angiogenesis, but is unsuitable for cancer therapy [10]. Roots derived from various *Bupleurum *species such as *B. chinense*, *B. scorzonerifolium*, *B. falcatum*, and *B. kaoi*, have been widely used in various medicinal decoctions. The content and proportion of the monomer SSs are extremely diverse in these medicinal materials. The concentration and composition of the SSs in the roots is even more complex when studied in combination with diverse planting and harvesting environments and different management methods.

**Figure 1:**
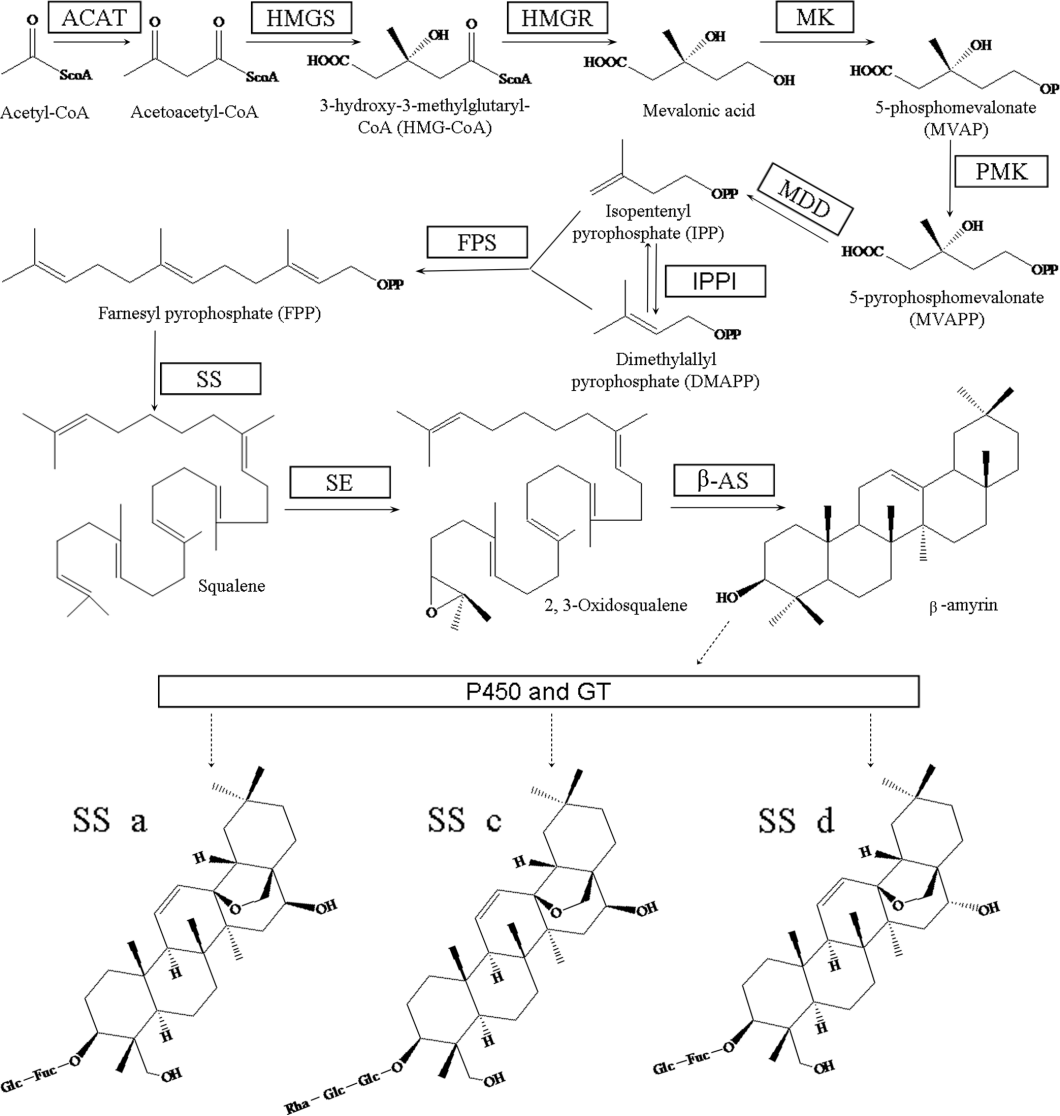
**Putative saikosaponin biosynthetic pathway in *B. chinense***. ACAT, acetyl-CoA acetyltransferase; β-AS, β-amyrin synthase; FPS, farnesyl diphosphate synthase; GT, glycosyltransferase; HMGR, HMG-CoA reductase; HMGS, HMG-CoA synthase; IPPI, isopentenyl diphosphate isomerase; MDD, mevalonate diphosphate decarboxylase; MK, mevalonate kinase; P450, cytochrome P450; PMK, phosphomevalonate kinase; SE, squalene epoxidase; SQS, squalene synthase; SS-a, saikosaponin-a; SS-c, saikosaponin-c; SS-d, saikosaponin-d.

The ability to control the SS content of these medicinal materials by up-regulating the genes involved in the biosynthesis of the different SS monomers or by using bio-engineering techniques would greatly improve their reliability. To be able to attempt this, an understanding of SS biosynthesis is required [13]. The putative SS biosynthetic pathway in *B. chinense *is shown in Figure 1. This pathway is based on previous studies on the biosynthetic pathway of other triterpene saponins in some plant species [14,15], as well as the pathways of SSs in *B. falcatum *[16] and *B. kaoi *[17]. The putative SS biosynthetic pathway initiates the isoprenoid pathway, mediates the cyclization of oxidosqualene, and then undergoes some modifications of oxidation, glycosylation, and other secondary transformations. Finally, the formation of the various monomer SSs is completed. The cDNA of β-amylase (β-AS) that catalyzes the formation of β-amyrin has been cloned in *B. kaoi *[18], and two different cDNAs that may encode different isoforms of β-AS have been cloned in *B. chinense*. Recently, these clones have been characterized in our laboratory (unpublished). In our previous studies, the cDNAs that encode enzymes involved upstream of the SS biosynthetic pathway in *B. chinense *have also been cloned [19-21]]. The biosynthetic pathways of the other saponins are not fully understood. Similarly, the reactions downstream of the SS biosynthetic pathway after the cyclization of β-amyrin remain largely unknown. One proposal involves oxidization/hydroxylation and glycosyl transfer catalyzed by specific cytochrome P450s and uridine diphosphate (UDP) glycosyltransferases (UGTs), respectively [14,22,23]]. To date, no *P450 *or *UGT *genes involved in SS biosynthesis have been identified in *B. chinense *or in other SS-producing plants. To the best of our knowledge, only a few *P450*s and *UGT*s have been verified to be involved in the biosynthesis of triterpenoid saponins [24-28]; recent reviews in [29,30]]. This situation is inconsistent with the fact that saponins are widely distributed in plants [31]. The biosynthetic pathway of the saponins still has to be clarified.

Studies have shown that the sequencing and analysis of expressed sequence tags (ESTs), combined with genetic and phytochemical methods are effective tools for discovering novel genes in non-model plants [32-34]]. Some genes involved in natural product biosynthetic pathways have been identified via EST analyses [24-26,28,35,36]]. The 454 pyrosequencing technique, with its advantages of throughput, read length, and accuracy, can greatly accelerate the discovery of novel genes in non-model organisms [37-39]]. Candidate genes involved in the metabolic pathways of natural products have been identified using the 454 pyrosequencing technique. These natural products usually have diverse and important functions in plant growth, and are also invaluable as pharmaceuticals and agrochemicals. Examples of such important natural products include triterpene saponins in American ginseng [15] and *Glycyrrhiza uralensis *[40], flavonoids in *Artemisia annua *[41], alkaloids in *Huperzia serrata *and *Phlegmariurus carinatus *[42], and cyanogenic glucosides in *Zygaena filipendulae *[43]. The specific functions of the candidate genes reported in the abovementioned studies are still being validated. Even so, the 454 pyrosequencing is still the preferred choice for novel gene discovery, especially for members of known gene families.

In the present study, 195, 088 high-quality (HQ) reads from a cDNA library of *B. chinense *were obtained using the Roche GS FLX Titanium platform. The reads were assembled into 24, 037 unique sequences comprising 22, 748 contigs and 1, 289 singletons. Only 864 ESTs were identical with those derived from the 3, 111 ESTs generated in our previous study from a *B. chinense *root cDNA library using the Sanger sequencing method [19] and the *Bupleurum *sequences from NCBI. A total of 246 *P450*s and 102 glycosyltransferases (*GT*s) including 49 *UGT*s were screened. The assembled full-length cDNAs of the *P450*s and *UGT*s were verified. Several partial cDNAs of the *P450*s and *UGT*s were extended to full length by 5' and/or 3' rapid amplification of cDNA ends (RACE). The candidate *P450*s and *UGT*s that may participate in SS biosynthesis were screened via methyl jasmonate (MeJA) inducibility and tissue-specific expression pattern experiments. These *P450*s and *UGT*s will be the targets of further research on SS biosynthesis.

## Results

### Sequencing and *de novo *assembly

**Figure 2:**
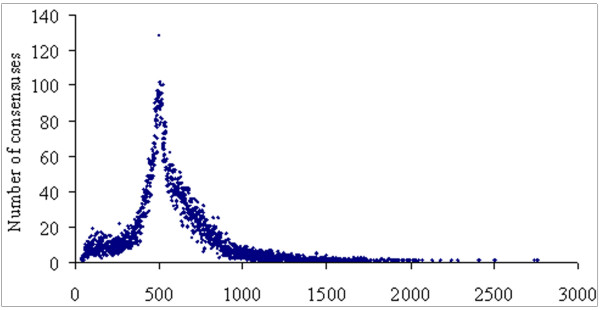
**Size distribution of *B. chinense *assembled consensuses (contigs and singletons)**.

A one-quarter plate run using the 454 GS FLX Titanium platform was carried out on the cDNA that was generated by SMART technology from the equivalent pooled total RNA from *B. chinense *roots, germinating seeds, and seedlings. A total of 195, 088 HQ reads with an average sequence length of 356 bp were obtained from 202, 126 raw reads after the initial quality filtering step [NCBI Short Read Archive, accession SRA039388]. The HQ reads were then assembled into 22, 748 contigs and 1, 289 singletons. The consensuses (contigs and singletons), equal to 13.8 Mb of sequence data, had an average length of 576 bp with a range of 43-2, 756 bp. The sequencing and assembly statistics are shown in Table 1. The size distribution of the consensuses is shown in Figure 2.

**Table 1:** Summary of *B. chinense *454 sequencing and assembly

Items	High-quality reads	Contigs	Singletons	Consensuses
Total number	195088	22748	1289	24037
Total bases (bp)	69381890	13402551	439796	13842347
Average length	355.7	589.2	341.2	575.9
Range of length	50-711	43-2756	51-572	43-2756

### Functional annotation

**Figure 3:**
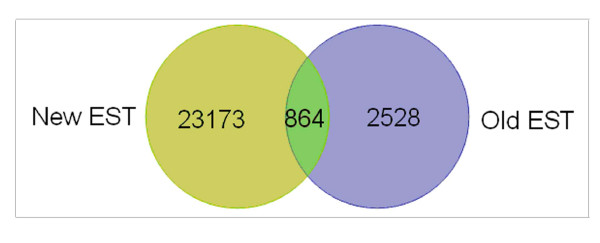
**Comparison of the 454 EST dataset and other EST datasets obtained for the genus *Bupleurum***. The overlapping section represents the ESTs that are present in the two datasets.

The assembled 24, 037 unique sequences were successively compared with the sequences in three major public protein databases (KEGG, Nr, and UniProt) using the basic local alignment search tool X (BLASTX) algorithm with an E-value cutoff of < 10^-10^. A total of 12, 649 unique sequences, accounting for 52.6% of the total unique sequences, were annotated (See additional file 1: Summary of the annotation of the 454 assembled unique *B. chinense *sequences). In our previous study, a total of 3, 111 cDNA clones derived from a *B. chinense *root cDNA library were 5' single-pass-sequenced using the Sanger sequencing method [19]. In the present study, the unique sequences obtained via 454 pyrosequencing were compared with the 3, 111 ESTs and with an additional 237 ESTs downloaded from GenBank. The unique sequences were also compared with the 44 *Bupleurum *protein-encoded sequences from GenBank. Only 864 unique sequences from the 454 dataset overlapped with other ESTs. Therefore, the 454 dataset must contain thousands of novel genes for the genus *Bupleurum *(Figure 3).

A sum of 10, 734 (44.7%) of the total unique sequences were further annotated based on their similarity with The Arabidopsis Information Resource (TAIR) proteins, before gene ontology (GO) terms were assigned. The categories of molecular function, biological process, and cellular component are shown in additional file 2: Functional annotations of the 454 unique sequences of *B. chinense *based on GO categories. A high percentage of the unique sequences were annotated to hydrolases, kinases, and transferases in the molecular function category. For the biological process category, a large number of genes were annotated to metabolic processes, response to abiotic or biotic stimulus, and response to stress. Hence, the 454 dataset should substantially aid the discovery of novel genes involved in the metabolism of SSs and other secondary natural products. A total of 10, 277 unique sequences were annotated using KEGG; 2, 849 of them were related to metabolism, 36 to the metabolism of terpenoids polyketides, and 101 to the biosynthesis of other secondary metabolites (see additional file 3: Summary of metabolic pathway assignments of the 454 assembled unique sequences based on KEGG).

### Candidate genes related to SS backbone biosynthesis

Similar to other triterpene glycoside pathways, the SS biosynthetic pathway is generally divided into three stages (Figure 1). The first stage is the formation of the isoprene units isopentenyl pyrophosphate (IPP) and dimethylallyl pyrophosphate (DMAPP), the second is the formation of the triterpene skeleton (β-amyrin), and the third stage is the modification of the skeleton. Based on our current knowledge, no definite sequence features can be used to identify the specific *P450*s and *UGT*s involved in the modification of the triterpene skeleton. Therefore, the unique sequences that were annotated as responsible for the formation of the SS backbones in the first two stages of the pathway were first screened. As shown in Table 2, except for mevalonate-5-diphosphate decarboxylase (MDD), the putative genes that encode all the enzymes for the biosynthesis of SS backbones were found. Triterpenoids are generally considered to be formed in the cytoplasm via the mevalonate (MVA) pathway. However, recent reports have demonstrated that the cytosolic MVA pathway and the plastidic methylerythritol phosphate (MEP) pathway may mutually communicate through the regulatory role of isopentenyl diphosphate isomerase (IPPI), which maintains the appropriate levels of IPP and DMAPP in the cytoplasm and plastids [44,45]. Consequently, three putative genes that encode the enzymes involved in the MEP pathway were also searched for and found in the 454 dataset. These genes were DXP reductoisomerase (DXPS; EC 1.1.1.267), MEP cytidylyltransferase (MEPCT; EC 2.7.7.60), and 4-hydroxy-3-methylbut-2-enyl-diphosphate synthase (GcpE/IspG; EC 1.17.7.1).

**Table 2:** Numbers of annotated unique sequences and 454 reads involved in saikosaponin skeleton biosynthesis

Enzyme code	Abbreviation	Enzyme name	Number of unique sequences	Number of 454 reads
2.3.1.9	ACAT	Acetyl-CoA acetyltransferase	4	10
2.3.3.10	HMGS	HMG-CoA synthase	2	6
1.1.1.34	HMGR	HMG-CoA reductase	1	2
2.7.1.36	MK	Mevalonate kinase	1	2
2.7.4.2	PMK	Phosphomevalonate kinase	1	1
4.1.1.33	MDD	Mevalonate-5-diphosphate decarboxylase	0	0
5.3.3.2	IPPI	Isopentenyl-PP isomerase	1	1
2.5.1.10	FPS	Farnesyl diphosphate synthase	2	27
2.5.1.21	SQS	Squalene synthase	6	30
1.14.99.7	SE	Squalene epoxidase	1	1
5.4.99.39	β-AS	β-Amyrin synthase	3	6

### Full-length cDNA cloning of *P450*s and *UGT*s

**Figure 4:**
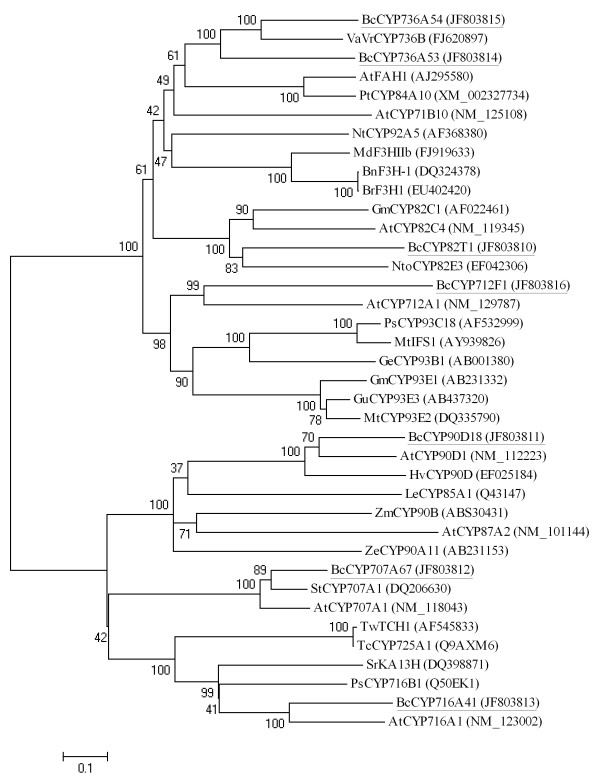
**Neighbor-joining bootstrap tree of P450 sequences from *B. chinense *and from other plant species**. Bootstrap values are shown as percentages. Sequences from *B. chinense *are underlined. The P450 sequences from *B. chinense *are the translated full-length sequences. At, *Arabidopsis thaliana*; Bc, *Bupleurum chinense*; Bn, *Brassica napus*; Br, *Brassica rapa *subsp. *Campestris*; Ge, *Glycyrrhiza echinata*; Gm, *Glycine max*; Gu, *Glycyrrhiza uralensis*; Hv, *Hordeum vulgare *subsp. *Vulgare*; Le, *Lycopersicon esculentum*; Lj, *Lotus japonicus*; Md, *Malus *x *domestica*; Mt, *Medicago truncatula*; Nt, *Nicotiana tabacum*; Nto, *Nicotiana tomentosiformis*; Ps, *Pisum sativum*; Pt, *Populus trichocarpa*; Sr, *Stevia rebaudiana*; St, *Solanum tuberosum*; Tc, *Taxus cuspidate*; Tw, *Taxus wallichiana *var. *chinensis*; VaVr, *Vitis arizonica *x *Vitis rupestris*; Ze, *Zinnia elegans*; Zm, *Zea mays*.

The *P450*s constitute one of the biggest gene families in plant genomes, accounting for more than 1% of the total gene annotations in each plant species [22]. In our unique sequences, a total of 239 contigs and 7 singletons were annotated as *P450*s accounting for about 1.02% of the 24, 037 unique sequences (see additional file 4: Putative P450 and GT genes in the 454 dataset). According to the best-hit description in the annotation databases, the P450s encoded by these unique sequences were classified into 30 families and 44 subfamilies; four of the P450s were unclassified (see additional file 5: Summary of family classification of the annotated P450s from the 454 assembled unique sequences). Two annotated full-length *P450*s were verified by reverse transcriptase polymerase chain reaction (RT-PCR). Three *P450*s that had single terminals in the 454 dataset, and two *P450*s derived from our previous cDNA library, were extended by RACE PCR. As a result, seven full-length *P450*s were obtained. The seven full-length *P450*s were further analyzed using BLASTX in NCBI. The deduced amino acid sequences were aligned with homologous *Arabidopsis thaliana *P450s and some function-identified P450s from other species. A neighbor-joining tree was constructed based on the alignment (Figure 4). The seven *P450*s were named by Nelson [46] as BcCYP82T1, BcCYP90D18, BcCYP707A67, BcCYP716A41, BcCYP736A53, BcCYP736A54, and BcCYP712F1.

GTs are a superfamily of enzymes in plants. GTs catalyze the transfer of sugar moieties from activated donor molecules to specific acceptor molecules, forming glycosidic bonds. Currently, there are 92 families and some non-classified sequences at the superfamily level http://www.cazy.org/GlycosylTransferases.html. Glycosylation is one of the major factors that determine the bioactivity and bioavailability of natural plant products, such as flavonoids and terpenoids.

**Figure 5:**
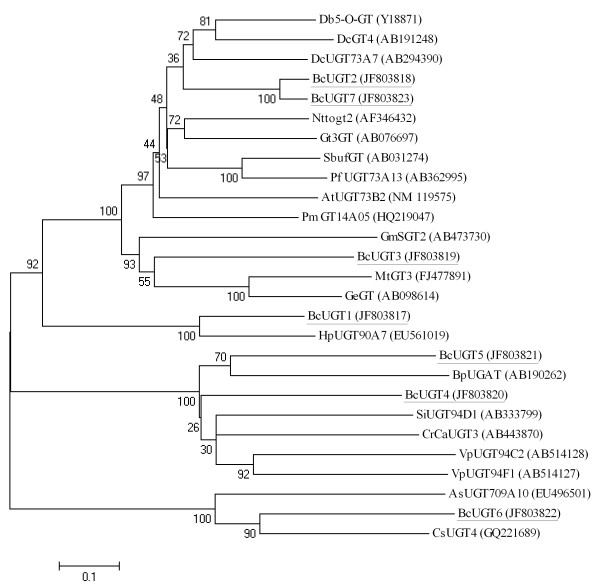
**Neighbor-joining bootstrap tree of UGT sequences from *B. chinense *and from other plant species**. Bootstrap values are shown as percentages. Sequences from *B. chinense *are underlined. The UGT sequences from *B. chinense *are the translated full-length sequences. As, *Avena strigosa*; At, *Arabidopsis thaliana*; Bc, *Bupleurum chinense*; Bp, *Bellis perennis*; Cr, *Catharanthus roseus*; Cs, *Citrus sinensis*; Db, *Dorotheanthus bellidiformis*; Dc, *Dianthus caryophyllus*; Ge, *Glycyrrhiza echinata*; Gm, *Glycine max*; Gt, *Gentiana triflora*; Hp, *Hieracium pilosella*; Mt, *Medicago truncatula*; Nt, *Nicotiana tabacum*; Pf, *Perilla frutescens*; Pm, *Pueraria montana *var. *lobata*; Sb, *Scutellaria baicalensis*; Si, *Sesamum indicum*; Vp, *Veronica persica*.

The GTs that are responsible for the glycosylation of natural products are members of the family 1 GTs, the UGTs [23]. In our unique sequences, there were 102 annotated *GT*s (see additional file 4: Putative P450 and GT genes in the 454 dataset) of which 49 were *UGT*s. All the *GT*s were classified into 14 categories according to the GO term assignment (see additional file 6: Classification of the candidate glycosyltransferase/glucosyltransferase genes). Two full-length *UGT*s were verified by RT-PCR. Four partial *UGT*s from the 454 dataset and one *UGT *derived from our previous cDNA library were extended by RACE PCR, resulting in seven full-length *UGT*s tentatively named *BcUGT1*, *BcUGT2*, *BcUGT3*, *BcUGT4*, *BcUGT5*, *BcUGT6*, and *BcUGT7*. The deduced amino acid sequences of these seven *UGT*s were aligned with some UGTs from other species, and a neighbor-joining tree was constructed based on the alignment (Figure 5). The UGTs from the other plant species were selected based on the BLASTX results for the seven UGTs cloned in the present study. Most of these UGTs were function identified. GmSGT2 (AB473730) and MtGT3 (FJ477891) were identified with functions in the biosynthesis of saponins. AsUGT709A10 (EU496501) and CsUGT4 (GQ221689) may be involved in the formation of triterpenoids and monoterpenoids, respectively, because both were registered in GenBank and relevant unpublished papers. The others were identified as flavonoid GTs. Although the functions of a *UGT *cannot be deduced from the sequence alignment alone, the functions of BcUGT3 (JF803819) and BcUGT6 (JF803822) were first verified in the course of SS-related UGT identification. Both of these UGTs had high sequence similarities with previously identified terpene GTs.

### Expression characteristics of *P450*s and *UGT*s

**Figure 6:**
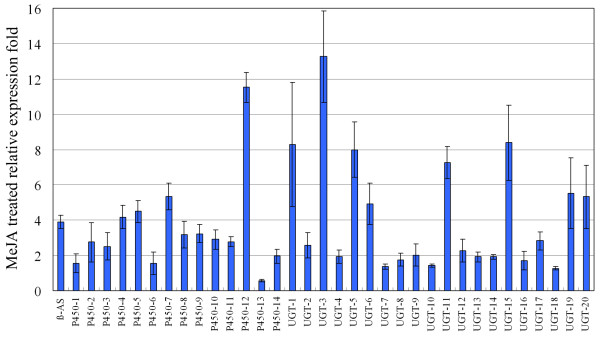
**Real-time PCR analysis of *β-AS*, *P450*s, and *UGT*s in MeJA-treated adventitious roots of *B. chinense***. The relative fold expressions for MeJA-treated roots and untreated controls are shown. β-AS, β-amyrin synthase. The corresponding unique sequences represented by P450-1-P450-14 and UGT-1-UGT-20 are listed in additional file 8.

The pentacyclic triterpenoid SSs are considered to be synthesized using β-amyrin as a substrate via a series of reactions thought to be catalyzed by *P450*s and *UGT*s. MeJA is known to induce the biosynthesis of many secondary metabolites, such as ginsenoside [47,48] and SSs [17,49]. MeJA also up-regulates metabolite-related enzyme genes [50]. The expression of genes involved in SS skeleton biosynthesis has not been studied in MeJA treatment experiments. However, the expression of *β-AS *is up-regulated with increased accumulation of SSs in the hairy roots of *B. falcatum *using altered culture media [16]. Therefore, in the present study, MeJA-treated adventitious roots of *B. chinense *were used to investigate the expression of *β-AS*, and to screen the putative *P450*s and *UGT*s involved in SS biosynthesis. Based on the classifications and read abundances, a total of 14 *P450*s and 20 *UGT*s were selected for MeJA inducibility analyses using real-time PCR with *actin *as the internal reference gene. All selected P450s belonged to the two clans, CYP71 and CYP85, that include all known triterpenes and sterol hydroxylases. As shown in Figure 6, *β-AS *expression increased about fourfold in MeJA-treated adventitious roots compared with in the control. All *P450*s and *UGT*s assayed were up-regulated by MeJA, whereas the elevated fold expressions of four *P450*s and eight *UGT*s were more than that of *β-AS*. Therefore, the four *P450*s (P450-4, P450-5, P450-7, and P450-12) and the eight *UGT*s (UGT-1, UGT-3, UGT-5, UGT-6, UGT-11, UGT-15, UGT-19, and UGT-20) were further assayed for their tissue-specific expression patterns.

**Figure 7:**
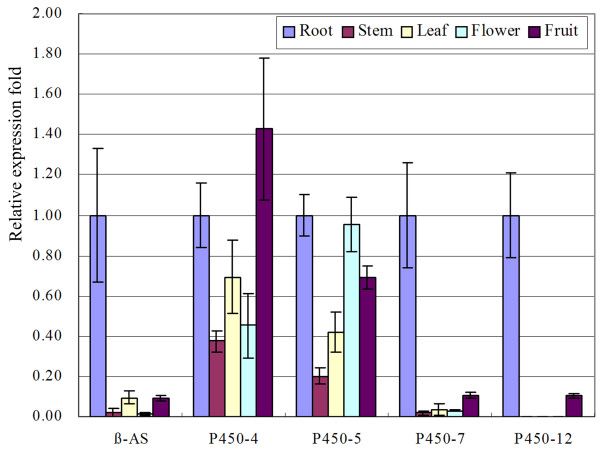
**Real-time PCR analysis of *P450*s in different plant tissues**. β-AS, β-amyrin synthase. The corresponding unique sequences represented by P450-4, P450-5, P450-7 and P450-12 are listed in additional file 8.

**Figure 8:**
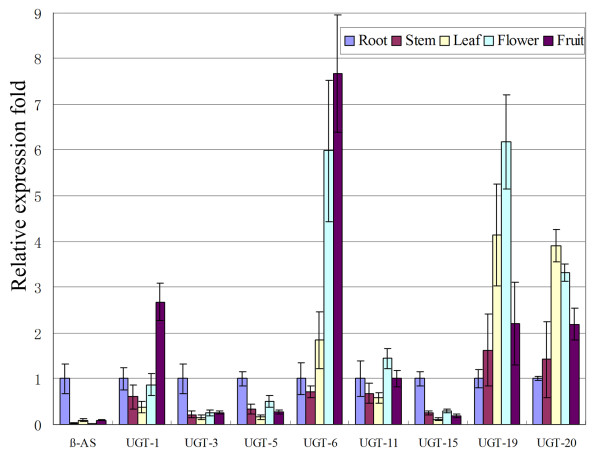
**Real-time PCR analysis of *UGT*s in different plant tissues**. β-AS, β-amyrin synthase. The corresponding unique sequences represented by UGT-1, UGT-3, UGT-5, UGT-6, UGT-11, UGT-15, UGT-19, and UGT-20 are listed in additional file 8

Five tissues, roots, stems, leaves, flowers and fruits, were used to analyze the tissue-specific expression patterns of the abovementioned four *P450*s and eight *UGT*s. Real-time PCR analysis was performed using *β-tubulin *as the internal reference gene. As shown in Figures 7 and 8, the expression patterns of two *P450*s (P450-7 and P450-12) and three *UGT*s (UGT-3, UGT-5, and UGT-15) showed strong similarities with that of *β-AS*. P450-12 was obtained from our previous cDNA library. P450-7, UGT-3, UGT-5, and UGT-15 were all obtained from the 454 dataset. The sequence similarity results suggest that UGT-3 and UGT-5 are members of the UGT85 family, whereas UGT-15 belongs to the UGT76 family. These *P450*s and *UGT*s can be considered as candidate genes encoding enzymes responsible for SS biosynthesis, and require further study.

## Discussion

The 454 pyrosequencing technology is regarded as a prime choice for novel gene discovery in non-model organisms. In the present study, this technology was applied with the main goal of identifying the *P450*s and *UGT*s involved in the biosynthesis of SSs in *B. chinense*. In previous reports, CYP93E1 from *Glycine max *was shown to hydroxylate β-amyrin and sophoradiol with the formation of olean-12-ene-3β, 24-diol, and soyasapogenol B, respectively [24]. CYP88D6 from *Glycyrrhiza uralensis *was identified as a β-amyrin 11-oxidase [25]. UGT73K1 and UGT71G1 from *Medicago truncatula *[26,27] and UGT74M1 from *Saponaria vaccaria *[28] have been identified to be involved in triterpene biosynthesis. Thus far, no *P450 *or *UGT *were identified in SS-producing plant species. All known triterpenes and sterol hydroxylases have been classified into the CYP71 and CYP85 clans [24,25,51,52]. In our 454 dataset, 114 unique sequences in 8 families and 52 unique sequences in 4 families belong to the CYP71 and CYP85 clans, respectively. Of these 49 were *UGT*s representing nine families, namely, UGT71, UGT72, UGT73, UGT74, UGT76, UGT84, UGT85, UGT91, and UGT94. Our data provide a promising opportunity for identifying the *P450*s and *UGT*s involved in SS biosynthesis. In the present study, 14 unique sequences of *P450*s and 20 *UGT*s were screened. Two *P450*s and three *UGT*s that may be involved in the biosynthesis of SSs, based on MeJA inducibility and tissue-specific expression patterns, were found. They are currently being identified by their heterologous expression in *Escherichia coli *or yeast, as well as by their overexpression and gene silencing in transgenic *B. chinense *plants. More candidate SS-related *P450*s and *UGT*s may be found among the annotated *P450*s and *UGT*s. Along with the identified *P450*s and *UGT*s, our results may also be helpful in revealing the formation mechanism of diverse monomer SSs and in elucidating other saponin biosynthetic pathways.

In the present study, the full-length cDNA clones of seven *P450*s and seven *UGT*s were obtained. Two of the *P450*s belong to the *CYP736 *family and the other five *P450*s belong to the *CYP82*, *CYP712*, *CYP90*, *CYP707*, and *CYP716 *families. The catalytic function of the CYP736 family is still unknown. Recent reports have shown that CYP736B in grapes may be involved in the host response to *Xylella fastidiosa *infection [53]. CYP736A34 in soybean is also highly co-expressed with genes involved in root and *Rhizobium*-induced nodule development [[54]; review in [55]]. CYP82 and CYP712 are part of the CYP71 clan family. Some members of the CYP82 family were found to mediate plant-specific alkaloid pathways, for example, CYP82E4 and CYP82E5v2 in tobacco were identified with nicotine *N*-demethylase activity [56,57]. Arabidopsis CYP82C2 and CYP82C4 are 8-methoxypsoralen hydroxylases that mediate modifications of toxic furanocoumarin [58]. However, a recent study has shown that CYP82G1 functions in the terpene pathway as a DMNT/TMTT (C11-homoterpene (E)-4, 8-dimethyl-1, 3, 7-nonatriene/C16-homoterpene (E, E)-4, 8, 12-trimethyltrideca-1, 3, 7, 11-tetraene) homoterpene synthase [59]. CYP712 and the CYP93s may catalyze successive steps in the same pathway(s) in different plants [55]. One of the CYP93s, CYP93E1, was found to participate in the triterpene pathway [24]. CYP90, CYP707, and CYP716 are part of the CYP85 clan family. CYP90 is the first family of CYPs required for brassinosteroid synthesis. CYP90Bs, -As, -Ds, and -Cs successively act in the brassinosteroid pathway [60,55]. The CYP707s inactivate ABA via 8'-hydroxylation to form phaseic acid, and thereby, play a key role in the regulation of ABA-mediated physiological processes [61]. The CYP716s do not have a known function, but their closest non-plant relatives, CYP26As, are involved in the hydroxylation of retinoic acid [55]. Based on sequence similarity, CYP716 was close to CYP725 in the neighbor-joining tree (Figure 4). A previous study using a broader range of plants also showed some overlap in CYP716 and CYP725. This overlap is evidence of the extensive divergence occurring within this subset of genes in the CYP85 clan. CYP725A has been shown to act on taxane diterpenoids [60]. However, it is still unclear whether these two families share similar functions. The seven *UGT*s for which full-length cDNAs were generated in the present study have sequence similarities with members of different *UGT *families. This finding implied that the *UGT*s identified in the present study may be members of these different *UGT *families. Based on the neighbor-joining tree (Figure 5), BcUGT3 was found to be close to members of the UGT73 family, in particular to GmSGT2 (UGT73P2), MtGT3 (UGT73F3) and GeGT (UGT73F1); BcUGT6 was close to a UGT709 member. BcUGT2 and BcUGT7 were also close to UGT73 members and to other UGTs without definite family ascriptions. BcUGT1 was close to a UGT90 member. Previous studies [62,63] have indicated that UGT73 and UGT90 belong to the same orthologous group, OG1 [63]. UGT73B2 was shown to exhibit flavonoid 7-*O*-glucosyltransferase activity [64], UGT73A7 has been reported to exhibit 4, 2', 4', 6'-tetrahydroxy chalcone 4'-glucosyltransferase activity [65], and UGT90A7 was shown to exhibit luteolin 4'/7-O-glucosyltransferase activity [66]. BcUGT4 and BcUGT5 may belong to the UGT94 family because they have sequence similarities with UGT94s. Previous studies have shown that UGT94D1 has UDP-glucose: sesaminol 2'-*O*-glucoside-*O*-glucosyltransferase activity and UGT94F1 has anthocyanin 3-O-glucoside-2''-O-glucosyltransferase activity [67]. Although the definite functions of the seven *P450*s and seven *UGT*s from *B. chinense *identified in the present study still have to be verified by further experiments, the isolation of their full-length cDNAs will be significant for elucidating their biofunctions in the growth and development of *B. chinense*.

The biosynthesis and regulation of bioactive components was the main focus of the present study on *B. chinense*. In addition to SSs isolated from members of the genus *Bupleurum *that exhibit pharmacological activity, several other groups of secondary metabolites with relevant biological activity have been characterized, for example, polysaccharides with anti-ulcer activity and lignans with anti-proliferative activity [68]. Genes involved in polysaccharides and lignans were searched for in the present 454 dataset. For example, enzymes encoded by genes related to polysaccharides include (1, 3)-beta-D-glucan synthase, alpha-1, 6-xylosyltransferase, alpha-(1, 4)-galacturonosyltransferase, xylan 1, 4-beta-xylosidase, etc. [69] and enzymes encoded by the genes related to lignans, are phenylalanine ammonia lyase, cinnamate 4-hydroxylase, 4-coumarate-CoA ligase, hydroxycinnamoyl CoA: shikimate/quinate hydroxycinnamoyltransferase, caffeoyl-CoA O-methyltransferase, isoeugenol synthase, and dirigent protein oxidase [70]. Therefore, the present 454 dataset is valuable not only in the exploration of genes involved in SS biosynthesis, but also for the discovery of genes involved in other bioactive secondary metabolites derived from the genus of *Bupleurum*. Additionally, the agronomical traits of *B. chinense*, such as drought resistance, have been investigated [71,72]. In our 454 dataset, 2, 933 and 3, 280 unique sequences were annotated as related to responses to abiotic or biotic stimulus and to stress, respectively. These annotations were based on the GO terms. These sequence data may be beneficial to further molecular studies on the stress response of *B. chinense*. Further, a total of 415 and 209 unique sequences were annotated with transcription factor activity and signal transduction, respectively. Some of these sequences may play roles in regulating SS metabolism and the stress response. These unique sequences deserve to be cloned and functionally analyzed in future studies.

Currently the 454 pyrosequencing technology is considered as a rapid and economical method to generate high-quantity sequence data. Although a large number of 454 reads were obtained by a quarter run in the present study, nearly a quarter of the ESTs from the Sanger-sequenced 3, 111 clones from our previous cDNA library were not sequenced. The different cDNA libraries (the Sanger sequenced root cDNA library and the 454 sequenced combined cDNA library with roots, seeds, and seedlings) and the fact that only the 5' end of the cDNA was sequenced in the Sanger sequencing may explain, to some extent, this difference. In some reports that compared 454 pyrosequencing and traditional Sanger sequencing, bias was found because of differences in the two sequencing methods [73]. Combinations of these two methods have been used in some studies: (1) to generate a high number of good-quality ESTs with improved clustering analysis and with more full-length sequences [73]; (2) to obtain a less biased method for the identification and diversity analysis of microbes and fungi [74,75]; and (3) to assemble genome sequences [76]. Recently, *Radix bupleuri *has aroused global interest, especially in Europe [review in [68]]. However, studies on the molecular biology of *Bupleurum *are still limited. More transcriptome data will facilitate a deeper understanding and enable the rapid development of *Radix bupleuri *applications.

## Conclusions

In the present study, a 454 dataset of *B. chinense *was analyzed. These data represent a substantial contribution to the functional genetic studies of *B. chinense*. The identification of enzymes involved in SS biosynthesis may enable the regulation and improvement of SS production levels in plants or in microbial hosts by metabolic engineering. Almost all of the known genes that encoded enzymes involved in the biosynthesis of the SS backbones were explored. A total of 246 *P450 *and 102 *GT *unique sequences containing 49 *UGT*s were obtained. These sequences will be invaluable to the elucidation of the SS biosynthetic pathway and to the exploration of the molecular mechanism underlying the biosynthesis of different monomer SSs. The full-length cDNAs of seven of the *P450*s and seven of the *UGT*s from our present 454 dataset and previous Sanger's sequencing data were cloned using the RACE method. This procedure may help in elucidating the functions of the *P450*s and *UGT*s. MeJA inducibility and tissue-specific expression pattern experiments were used to screen two *P450*s and three *UGT*s that may be involved in SS biosynthesis.

## Methods

### Plant material and adventitious root preparation

The roots of one-year old plants of "Zhongchai No. 1", a mass-selected cultivar of *B. chinense *field-grown in IMPLAD, were collected during the flowering stage because more SSs were found to be contained in the roots during this period [77]. Further, a previous study showed that the SS-d and SS-c content significantly changed in germinating seeds and the content of SS-d peaked on the fourth day [78]. Hence, to acquire a high number of unique candidate genes involved in SS biosynthesis the experimental material used in the present study was 4-day geminating seeds, 12-day seedlings, and the roots of one-year-old Zhongchai No. 1 plants during flowering. The germination was performed in germination boxes under 25°C/15°C, 8L/16D conditions. Before germination, the seeds were soaked for 24 h in tap water, which was changed four times. After harvest, all materials were immediately frozen in liquid nitrogen and stored in a -80°C freezer for RNA extraction.

To analyze the MeJA inducibility of P450s and UGTs, the adventitious roots of Zhongchai No. 1 were cultivated as described earlier [79]. Similar to the results of our previous experiment, the SS content was approximately doubled in 8 h MeJA-treated (200 μM, dissolved in ethanol) adventitious roots of *B. chinense*, assayed by high performance liquid chromatography [80]. MeJA (200 μM) was then added to the cultivation media; an equal quantity of ethanol was used as the control. After 8 h, the treated and control adventitious roots were collected and immediately stored in liquid nitrogen for RNA extraction. For the tissue-specific expression pattern experiments, five tissues (roots, stems, leaves, flowers, and fruits) were collected and similarly restored as described in our previous report [81].

### RNA extraction, cDNA library construction and 454 sequencing

Total RNA was isolated using an RNA purification kit (Norgen Biotek Corp., ON, Canada). RNA purity and degradation were checked on 1% agarose gels. Equivalent RNAs from roots, germinating seeds, and seedlings were pooled. Approximately 1 μg of RNA was reverse transcribed using a Super SMART™ PCR cDNA synthesis kit (Clontech Laboratories, Inc., Mountain View, CA, USA). This kit was used in combination with a modified poly (T) primer to overcome the limitation of long poly (A/T) tails in cDNA for the 454 sequencing [15]. Double-stranded (ds) cDNA was synthesized using an Advantage^® ^2 PCR kit (Clontech Laboratories, Inc.) and was then digested overnight with *Bsg*I (New England Biolabs, Ipswich, MA, USA). The ds cDNA was finally purified using a PureLink™ PCR purification kit (Invitrogen Life Science Technologies, Carlsbad, CA, USA). About 5 μg of ds cDNA was sent to the Roche 454 Company (Branford, CT, USA) for pyrosequencing using a GS FLX titanium kit.

### The 454 EST assembly and annotation

A pretreatment process that involved trimming the adapter and poly (A/T), as well as removing short sequence (< 50 bp) and low quality files (quality score threshold = 20) was performed. The Mira 3.0.5 software was used for sequence assembly using the default parameters. Reads that did not fit into a contig were defined as singletons. A total of 195, 088 HQ reads assembled in 22, 748 contigs and 1, 289 singletons were finally obtained for further functional annotation with the BLASTX program. The databases KEGG http://www.genome.jp/kegg/, Nr http://www.ncbi.nlm.nih.gov, and UniProt http://www.expasy.ch/sprot were used for the search. GO terms were assigned to the assembled unique genes based on similarities with *A. thaliana *protein sequences (TAIR9, http://www.arabidopsis.org). A cut-off value of *E *< 1.0^-10 ^was used in all BLASTX searches. The newly assembled unique genes were compared against the *Bupleurum *EST/protein encoding sequences in GenBank. The ESTs were derived from a *B. chinense *root cDNA library that was sequenced by our group using an ABI 3730 sequencer [19].

### Searching for candidate genes involved SS biosynthesis

The candidate genes *HMGR*, *IPPI*, *FPS*, *SQS*, *SE*, *β-AS*, *P450*, and *UGT *that are known to be involved in the biosynthesis of SSs were searched for within the text of the annotated unique genes based on their gene names and synonyms. The items from different annotation databases that were repeated were manually erased.

### Full-length cDNA verification and cloning of P450s and UGTs

The assembled full-length P450 and UGT cDNA sequences were verified by RT-PCR. Some partial sequences were extended to full length using 5' and/or 3' RACE. The amino acid sequence alignments of the full-length P450 and UGT cDNAs were created in MEGA 4 using CLUSTALW with default settings. Phylogenetic neighbor-joining trees were constructed and bootstrapped with 1000 iterations in MEGA 4. Corresponding sequences from other plants with the most similarity to each full-length P450 and UGT cDNA (obtained both in our present and previous studies) [19] were identified and downloaded from GenBank. These sequences were used for the alignments and tree constructions.

### Real-time PCR analysis

*Actin *was chosen as the internal reference gene for the real-time PCR gene expression analysis of MeJA-treated *B. kaoi *[17]. Similar to our previous report, *β-tubulin *was the most suitable reference gene for the real-time PCR analysis of tissue-specific gene expression patterns in *B. chinense *[81]. According to one of our previous experiments (data unpublished), *EF1α *was also a suitable internal reference gene for real-time PCR analysis in MeJA-treated *B. chinense*. In the present study, the suitability of *actin*, *β-tubulin*, and *EF1α *as internal reference genes in the MeJA-treated adventitious roots of *B. chinense *was first determined. Based on the results (see additional file 7: Screening of internal reference genes for real-time PCR analysis of MeJA inducibility), *actin *was selected as the internal reference gene for the MeJA inducibility experiment. For the tissue-specific expression pattern experiment, *β-tubulin *was selected as the internal reference gene based on our previous research [81]. All real-time PCR analyses were performed according to our previous report [81] with the following modifications: the RNA was extracted using an RNA purification kit (Norgen Biotek Corp.); the quantification of cDNA was performed on a NanoDrop ND 2000 spectrophotometer (Thermo Fisher Scientific Inc., Wilmington, DE, USA); and a SYBR^® ^PrimeScript^® ^RT-PCR kit II (Perfect Real-Time; TAKARA Bio Inc., Shiga, Japan) was used for the reverse transcription and real-time PCR. Two-step amplification conditions were used: 3 min at 95°C, 40 cycles of 30 s at 95°C, and 20 s at 58°C. For the analyses of the tissue-specific expression patterns, the expression in the root was arbitrarily chosen as the calibrator for each gene. For the MeJA inducibility experiment, the expression of each gene in the control was used as the calibrator. All primers used are listed in additional file 8: The primers used in the present study.

## Authors' contributions

CS conceived the study, designed and built the cDNA library, designed the primers for the RACE and the real-time PCR, participated in the data analyses, as well as drafted the manuscript. JZ performed part of the RACE and the real-time PCR experiments. JHW initiated the project, helped conceive the study, and revised the manuscript. SLC helped conceive the study and revised the manuscript. YL and CXX helped collect the sample and analyze the data. JSX performed part of the RACE experiment. YJ helped assay the content of SSs by HPLC in the pre-experiment. ZHG helped build the cDNA library. HJC helped draw the chemical structures. CMY and ZZ helped conceive the study. YHX helped analyze the gene functions. All authors read and approved the final manuscript.

## Supplementary Material

Additional file 1 **Summary of the annotation of the 454 assembled unique *B. chinense *sequences**. The annotations were obtained by comparing the assembled sequences with sequences from KEGG, Nr, and UniProt (*E *< 1 × 10^-10^). Click here for file

Additional file 2 **Functional annotations of the 454 unique sequences of *B. chinense *based on GO categories**. The annotations were obtained by assigning the 454 assembled unique sequences to the GO categories of molecular function, biological process, and cellular component based on their similarities with *A. thaliana *protein sequences (TAIR9, http://www.arabidopsis.org). A cut-off value of *E *< 1.0^-10 ^was used. Click here for file

Additional file 3 **Summary of metabolic pathway assignments of the 454 assembled unique sequences based on KEGG**. The numbers of 454 assembled unique sequences that were assigned into different metabolism categories based on KEGG are shown in a bar chart. Click here for file

Additional file 4 **Putative P450 and GT genes in the 454 dataset**. The 454 assembled unique sequences that were annotated as P450 and GT genes by comparing the assembled sequences with sequences from KEGG, Nr, and UniProt (*E *< 1 × 10^-10^) were manual identified and listed. Click here for file

Additional file 5 **Summary of family classification of the annotated P450s from the 454 assembled unique sequences**. The number of annotated 454 unique sequences and reads of *B. chinense *encoding P450s that belong to different families and subfamilies are listed. Families belong to the CYP71 clan are shown in red, and families belong to the CYP85 clan are shown in blue. Click here for file

Additional file 6 **Classification of the candidate glycosyltransferase/glucosyltransferase genes**. The assembled 454 unique sequences that were annotated as genes with various glycosyltransferase/glucosyltransferase activities were classified and listed. The classification was obtained by comparing annotated glycosyltransferase/glucosyltransferase genes from the 454 dataset with *A. thaliana *protein sequences (TAIR9, http://www.arabidopsis.org). Click here for file

Additional file 7 **Screening of internal reference genes for real-time PCR analysis of MeJA inducibility**. The RNA transcription levels of *actin*, *β-tubulin*, and *EF1α *in the MeJA-treated and control adventitious roots of *B. chinense *were assayed by real-time PCR and are presented as Ct values. Click here for file

Additional file 8 **The primers used in the present study**. All primers used for full-length cDNA cloning and real-time PCR analysis of P450s and UGTs in the present study are listed. Click here for file
