# Supplementary material for: Transcriptome analysis of Bupleurum chinense focusing on genes involved in the biosynthesis of saikosaponins
Source: BMC Genomics. 2011 Nov 2;12:539. doi: 10.1186/1471-2164-12-539 (PMC3219613; doi:10.1186/1471-2164-12-539)
Supplement: Additional file 1 — Summary of the annotation of the 454 assembled unique B. chinense sequences. The annotations were obtained by comparing the assembled sequences with sequences from KEGG, Nr, and UniProt (E < 1 × 10^-10^). [file 1471-2164-12-539-S1.DOC]

**Additional File 1 - Summary of the annotation of the 454 assembled unique *B. chinense* sequences. The annotations were obtained by comparing the assembled sequences with sequences from KEGG, Nr, and UniProt (*E* <1 × 10^-10^).**

| **Annotation database** | **Annotation number** | **Annotation percentage (%)** |
| --- | --- | --- |
| **KEGG** | 10,277 | 42.8 |
| **Nr** | 10,409 | 43.3 |
| **UniProt** | 9,453 | 39.3 |
| **Total** | 12,649 | 52.6 |
