# Supplementary material for: Transcriptome analysis of Bupleurum chinense focusing on genes involved in the biosynthesis of saikosaponins
Source: BMC Genomics. 2011 Nov 2;12:539. doi: 10.1186/1471-2164-12-539 (PMC3219613; doi:10.1186/1471-2164-12-539)
Supplement: Additional file 2 — Functional annotations of the 454 unique sequences of B. chinense based on GO categories. The annotations were obtained by assigning the 454 assembled unique sequences to the GO categories of molecular function, biological process, and cellular component based on their similarities with A. thaliana protein sequences (TAIR9, http://www.arabidopsis.org). A cut-off value of E < 1.0-10 was used. [file 1471-2164-12-539-S2.DOC]

**Additional File 2 –Functional annotations of the 454 unique sequences of *B. chinense* based on GO categories.** The annotations were obtained by assigning the 454 assembled unique sequences to the GO categories of molecular function, biological process, and cellular component based on their similarities with *A. thaliana* protein sequences (TAIR9, [http://www.arabidopsis.org](http://www.arabidopsis.org/)). A cut-off value of *E* <1.0^-10^ was used.

| DNA or RNA binding | hydrolase activity | kinase activity | nucleic acid binding | nucleotide binding | other binding | other enzyme activity | other molecular functions | protein binding | receptor binding or activity | structural molecule activity | transcription factor activity | transferase activity | transporter activity | unknown molecular functions | cell organization and biogenesis | developmental processes | DNA or RNA metabolism | electron transport or energy pathways | other biological processes | other cellular processes | other metabolic processes | protein metabolism | response to abiotic or biotic stimulus | response to stress | signal transduction | transcription | transport | unknown biological processes | cell wall | chloroplast | cytosol | ER | extracellular | Golgi apparatus | mitochondria | nucleus | other cellular components | other cytoplasmic components | other intracellular components | other membranes | plasma membrane | plastid | ribosome | unknown cellular components |
| --- | --- | --- | --- | --- | --- | --- | --- | --- | --- | --- | --- | --- | --- | --- | --- | --- | --- | --- | --- | --- | --- | --- | --- | --- | --- | --- | --- | --- | --- | --- | --- | --- | --- | --- | --- | --- | --- | --- | --- | --- | --- | --- | --- | --- |
| **Molecular Function** | | | | | | | | | | | | | | | **Biological Process** | | | | | | | | | | | | | | **Cellular Component** | | | | | | | | | | | | | | | |
