# Supplementary material for: Transcriptome analysis of Bupleurum chinense focusing on genes involved in the biosynthesis of saikosaponins
Source: BMC Genomics. 2011 Nov 2;12:539. doi: 10.1186/1471-2164-12-539 (PMC3219613; doi:10.1186/1471-2164-12-539)
Supplement: Additional file 3 — Summary of metabolic pathway assignments of the 454 assembled unique sequences based on KEGG. The numbers of 454 assembled unique sequences that were assigned into different metabolism categories based on KEGG are shown in a bar chart. [file 1471-2164-12-539-S3.DOC]

**Additional File 3 -** **Summary of metabolic pathway assignments of the 454 assembled unique sequences based on KEGG.** The numbers of 454 assembled unique sequences that were assigned into different metabolism categories based on KEGG are shown in a bar chart.
