# Supplementary material for: Transcriptome analysis of Bupleurum chinense focusing on genes involved in the biosynthesis of saikosaponins
Source: BMC Genomics. 2011 Nov 2;12:539. doi: 10.1186/1471-2164-12-539 (PMC3219613; doi:10.1186/1471-2164-12-539)
Supplement: Additional file 5 — Summary of family classification of the annotated P450s from the 454 assembled unique sequences. The number of annotated 454 unique sequences and reads of B. chinense encoding P450s that belong to different families and subfamilies are listed. Families belong to the CYP71 clan are shown in red, and families belong to the CYP85 clan are shown in blue. [file 1471-2164-12-539-S5.DOC]

**Additional File 5 –Summary of family classification of the annotated P450s from the 454 assembled unique sequences.** The number of annotated 454 unique sequences and reads of *B. chinense* encoding P450s that belong to different families and subfamilies are listed. Families belong to the CYP71 clan are shown in red, and families belong to the CYP85 clan are shown in blue.

| **P450 family** | **Subfamily No.** | **Unique gene No.** | **454 reads No.** |
| --- | --- | --- | --- |
| CYP71 | 4 | 84 | 697 |
| CYP72 | 2 | 31 | 178 |
| CYP716 | 3 | 27 | 414 |
| CYP87 | 2 | 15 | 170 |
| CYP749 | 1 | 10 | 57 |
| CYP83 | 1 | 10 | 39 |
| CYP94 | 1 | 9 | 85 |
| CYP76 | 4 | 8 | 48 |
| CYP707 | 1 | 7 | 78 |
| CYP704 | 2 | 5 | 31 |
| CYP706 | 3 | 5 | 12 |
| CYP92 | 1 | 5 | 19 |
| CYP82 | 2 | 4 | 25 |
| CYP736 | 1 | 3 | 8 |
| CYP90 | 1 | 3 | 13 |
| CYP97 | 1 | 2 | 6 |
| CYP3 | 1 | 1 | 2 |
| CYP4 | 1 | 1 | 4 |
| CYP509 | 1 | 1 | 5 |
| CYP51 | 1 | 1 | 3 |
| CYP710 | 1 | 1 | 2 |
| CYP711 | 1 | 1 | 2 |
| CYP712 | 1 | 1 | 8 |
| CYP714 | 1 | 1 | 2 |
| CYP721 | 1 | 1 | 2 |
| CYP728 | 1 | 1 | 2 |
| CYP73 | 1 | 1 | 2 |
| CYP80 | 1 | 1 | 3 |
| CYP86 | 1 | 1 | 2 |
| CYP93 | 1 | 1 | 2 |
| Unclassified |  | 4 | 12 |
| Total (30) | 44 | 246 | 1933 |
