# Supplementary material for: Transcriptome analysis of Bupleurum chinense focusing on genes involved in the biosynthesis of saikosaponins
Source: BMC Genomics. 2011 Nov 2;12:539. doi: 10.1186/1471-2164-12-539 (PMC3219613; doi:10.1186/1471-2164-12-539)
Supplement: Additional file 6 — Classification of the candidate glycosyltransferase/glucosyltransferase genes. The assembled 454 unique sequences that were annotated as genes with various glycosyltransferase/glucosyltransferase activities were classified and listed. The classification was obtained by comparing annotated glycosyltransferase/glucosyltransferase genes from the 454 dataset with A. thaliana protein sequences (TAIR9, http://www.arabidopsis.org). [file 1471-2164-12-539-S6.DOC]

**Additional file 6 - Classification of the candidate glycosyltransferase/glucosyltransferase genes. The assembled 454 unique sequences that were annotated as genes with various glycosyltransferase/glucosyltransferase activities were classified and listed. The classification was obtained by comparing annotated glycosyltransferase/glucosyltransferase genes from the 454 dataset with *A. thaliana* protein sequences (TAIR9,** [**http://www.arabidopsis.org**](http://www.arabidopsis.org/)**).**

| **GO terms for glycosyltransferase/glucosyltransferase** | **Unique sequence No.** | **Unique sequence** |
| --- | --- | --- |
| acetylglucosaminyltransferase activity | 1 | CH_c9888 |
| cellulose synthase activity | 7 | CH_c14779；CH_c2962；CH_c4260；CH_c7379；CH_c7902；CH_rep_c1261；CH_rep_c7115 |
| ceramide glucosyltransferase activity | 1 | CH_c7864 |
| cis-zeatin O-beta-D-glucosyltransferase activity | 2 | CH_c17593；CH_c9545 |
| dolichyl-diphosphooligosaccharide-protein glycotransferase activity | 6 | CH_c4810；CH_rep_c1053；CH_rep_c21341；CH_rep_c2304；CH_rep_c321；CH_rep_c5435 |
| glucuronosyltransferase activity | 8 | CH_c10436；CH_c14589；CH_c17593；CH_c3783；CH_c9545；CH_rep_c1716；CH_rep_c7668；CH_rep_c9908 |
| oligosaccharyl transferase activity | 4 | CH_c4810；CH_rep_c3350；CH_rep_c4439；CH_rep_c5435 |
| polygalacturonate 4-alpha-galacturonosyltransferase activity | 8 | CH_c11994；CH_c12364；CH_c14957；CH_c16519；CH_c4838；CH_c5762；CH_rep_c11455；CH_rep_c7465 |
| sinapate 1-glucosyltransferase activity | 2 | CH_c10468；CH_rep_c8491 |
| transferase activity, transferring glycosyl groups | 72 | CH_c10436；CH_c10468；CH_c11113；CH_c11994；CH_c12364；CH_c12893；CH_c14314；CH_c14589；CH_c14779；CH_c14957；CH_c16306；CH_c16359；CH_c16470；CH_c16519；CH_c17349；CH_c17593；CH_c1973；CH_c2962；CH_c3783；CH_c4260；CH_c4814；CH_c4838；CH_c5762；CH_c6487；CH_c6835；CH_c7183；CH_c7208；CH_c7379；CH_c7902；CH_c8483；CH_c9545；CH_c9862；CH_c9888；CH_rep_c11455；CH_rep_c1261；CH_rep_c1307；CH_rep_c15285；CH_rep_c1532；CH_rep_c1580；CH_rep_c16141；CH_rep_c1716；CH_rep_c18069；CH_rep_c18628；CH_rep_c18805；CH_rep_c19239；CH_rep_c19606；CH_rep_c1985；CH_rep_c20178；CH_rep_c21665；CH_rep_c2218；CH_rep_c23511；CH_rep_c2715；CH_rep_c2788；CH_rep_c2901；CH_rep_c3714；CH_rep_c39；CH_rep_c4581；CH_rep_c4656；CH_rep_c4954；CH_rep_c5711；CH_rep_c703；CH_rep_c7115；CH_rep_c7465；CH_rep_c7668；CH_rep_c7696；CH_rep_c8224；CH_rep_c836；CH_rep_c8491；CH_rep_c8604；CH_rep_c8896；CH_rep_c9346；CH_rep_c9908 |
| transferase activity, transferring hexosyl groups | 21 | CH_c10468；CH_c11994；CH_c12364；CH_c12880；CH_c14077；CH_c14957；CH_c16249；CH_c16470；CH_c16519；CH_c17349；CH_c17593；CH_c4838；CH_c6835；CH_c8483；CH_c9376；CH_c9545；CH_rep_c1532；CH_rep_c1580；CH_rep_c16141；CH_rep_c4126；CH_rep_c7465 |
| trans-zeatin O-beta-D-glucosyltransferase activity | 2 | CH_c17593；CH_c9545 |
| UDP-glucosyltransferase activity | 13 | CH_c11113；CH_c16249；CH_c16470；CH_c5395；CH_c6487；CH_c6835；CH_c7208；CH_c9376；CH_rep_c1532；CH_rep_c16141；CH_rep_c4126；CH_rep_c7696；CH_rep_c8491 |
| UDP-glycosyltransferase activity | 49 | CH_c10436；CH_c10468；CH_c11113；CH_c12880；CH_c12893；CH_c14077；CH_c14314；CH_c14589；CH_c16249；CH_c16306；CH_c16359；CH_c16470；CH_c17593；CH_c1973；CH_c3783；CH_c5395；CH_c6487；CH_c6835；CH_c7183；CH_c7208；CH_c9376；CH_c9545；CH_c9862；CH_rep_c13408；CH_rep_c15285；CH_rep_c1532；CH_rep_c1580；CH_rep_c16141；CH_rep_c1716；CH_rep_c18069；CH_rep_c18237；CH_rep_c18628；CH_rep_c18805；CH_rep_c19606；CH_rep_c1985；CH_rep_c20178；CH_rep_c21665；CH_rep_c21806；CH_rep_c2218；CH_rep_c3714；CH_rep_c39；CH_rep_c4126；CH_rep_c7668；CH_rep_c7696；CH_rep_c8224；CH_rep_c8491；CH_rep_c8604；CH_rep_c8896；CH_rep_c9908 |
