# Supplementary figures and images for: Transcriptome analysis of Bupleurum chinense focusing on genes involved in the biosynthesis of saikosaponins
Source: BMC Genomics. 2011 Nov 2;12:539. doi: 10.1186/1471-2164-12-539 (PMC3219613; doi:10.1186/1471-2164-12-539)

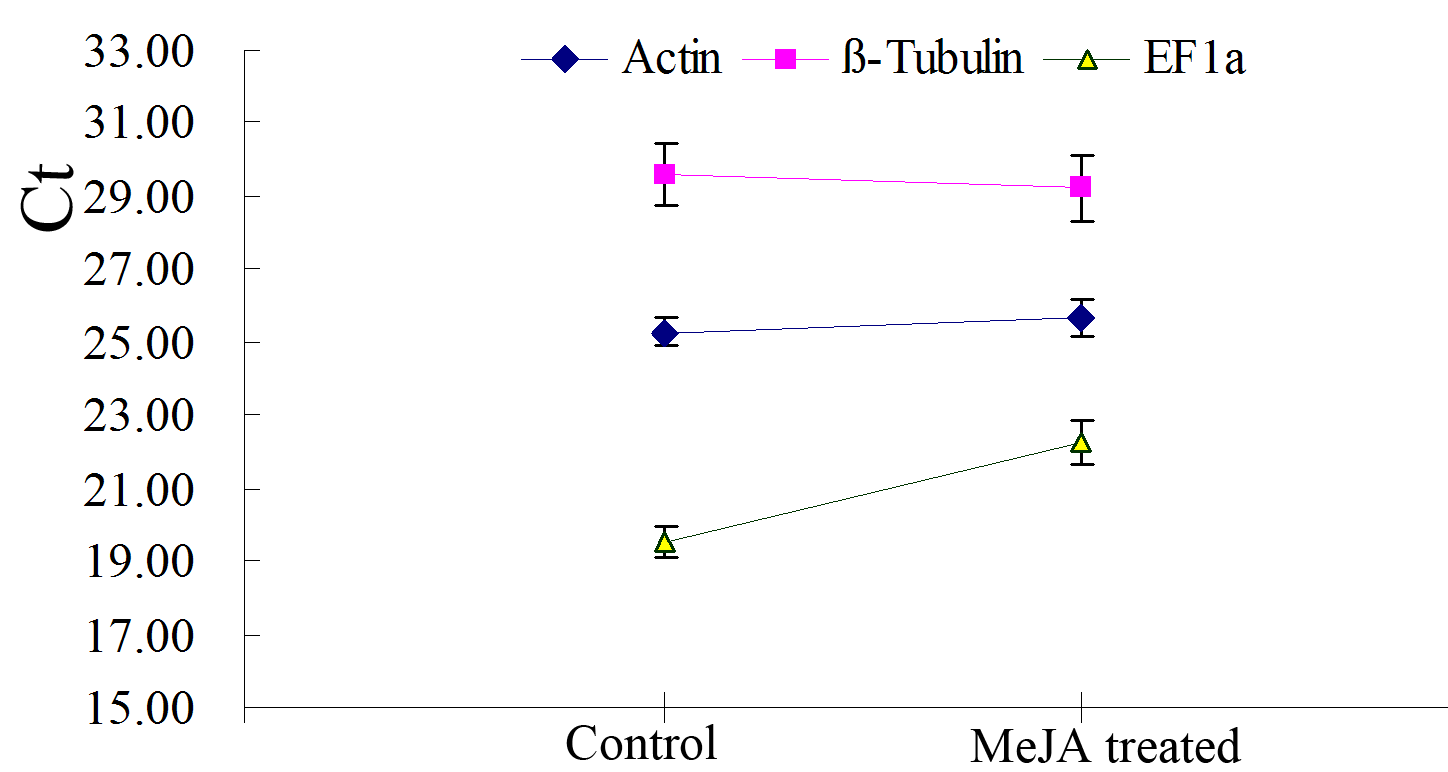

Supplement: Additional file 7 — Screening of internal reference genes for real-time PCR analysis of MeJA inducibility. The RNA transcription levels of actin, β-tubulin, and EF1α in the MeJA-treated and control adventitious roots of B. chinense were assayed by real-time PCR and are presented as Ct values. [file 1471-2164-12-539-S7.TIFF]
